# High prevalence and incidence of curable sexually transmitted infections among young women using oral HIV pre-exposure prophylaxis in sub-Saharan Africa: results from the INSIGHT Cohort study

**DOI:** 10.1136/sextrans-2025-056625

**Published:** 2025-12-25

**Authors:** Irene Mukui, Sue Peacock, Deborah Donnell, Brenda Gati-Mirembe, Meighan Krows, Elizabeth A Bukusi, Loveness Imaan, Philip Kotze, Katherine Margaret Gill, Pippa Macdonald, Cheryl Louw, Manjeetha Jaggernath, Anita Marais, Remco P H Peters, Sinead Delany-Moretlwe, Amy Ward, Phillip du Preez, Margaret Kasaro, Monica Gandhi, Renee Heffron, Connie Celum

**Affiliations:** 1 Department of Global Health, University of Washington, Seattle, Washington, USA; 2 Fred Hutchinson Cancer Center, Seattle, Washington, USA; 3 Makerere University-Johns Hopkins University Research Collaboration, Kampala, Central Region, Uganda; 4 Center for Microbiology Research, Kenya Medical Research Institute, Nairobi, Kenya; 5 Johns Hopkins Research Project-Kamuzu University of Health Sciences, Blantyre, Malawi; 6 Qhakaza Mbokodo Research Clinic, Ladysmith, South Africa; 7 Desmond Tutu HIV Centre, Institute of Infectious Disease and Molecular Medicine, Faculty of Health Sciences, University of Cape Town, Cape Town, Western Cape, South Africa; 8 Madibeng Centre for Research, Brits, South Africa; 9 Wits Maternal Adolescent and Child Health Research, Durban, South Africa; 10 Perinatal HIV Research Unit HIV Prevention Clinical Research Site, University of the Witwatersrand, Soweto, South Africa; 11 Foundation for Professional Development, Research Unit, East London, Pretoria, South Africa; 12 Wits RHI, University of the Witwatersrand, Hillbrow, Johannesburg, South Africa; 13 Vuka Research Clinic, Department of Medicine, University of Cape Town, Cape Town, South Africa; 14 University of North Carolina Global Projects Zambia, Lusaka, Zambia; 15 Department of Medicine, University of California, San Francisco, San Francisco, California, USA; 16 Department of Medicine, University of Alabama, Birmingham, Alabama, USA; 17 Department of Epidemiology, University of Washington, Seattle, Washington, USA

**Keywords:** Prevalence, Reproductive Tract Infections, AFRICA, Pre-Exposure Prophylaxis, WOMEN

## Abstract

**Abstract:**

**Background:**

HIV pre-exposure prophylaxis (PrEP) programmes in Africa reach young women at risk of sexually transmitted infections (STIs). We evaluated curable STI prevalence, incidence and risk factors among women initiating PrEP.

**Methods:**

From August to December 2022, sexually active women aged 16–30 years from 15 South African sites, and one site each in Eswatini, Kenya, Malawi, Uganda and Zambia were enrolled into the INSIGHT cohort and offered oral emtricitabine/tenofovir PrEP with follow-up at 1, 3 and 6 months. At each visit, STI symptoms were assessed and treatment provided based on syndromic management or diagnostic testing. Diagnostic tests included nucleic acid amplification for *Chlamydia trachomatis* and *Neisseria gonorrhoeae*, the rapid OSOM for *Trichomonas vaginalis* at enrolment and month 6, and serological testing for syphilis at enrolment using rapid plasma reagin with confirmatory *Treponema pallidum* particle agglutination. Prevalence and incidence of each STI were calculated, and predictors assessed using multivariable regression.

**Results:**

Of 3087 participants offered daily oral PrEP with a median age of 23 (IQR 21–27), 3011 had STI results and 30.9% had one or more STIs, with 15.7% reporting symptoms. The prevalence of *C. trachomatis*, *N. gonorrhoeae*, *T. vaginalis* and syphilis was 20.8%, 6.8%, 6.0% and 4.4%, respectively. The incidence of one or more STIs (*C. trachomatis*, *N. gonorrhoeae* or *T. vaginalis*) was 49.3/100 person-years (95% CI 45.3 to 53.4) with 12.7% reporting symptoms. The incidence of *C. trachomatis* was 30.6/100 person-years (95% CI 27.5 to 33.7), *N. gonorrhoeae* 10.8/100 person-years (95% CI 9.0 to 12.6) and *T. vaginalis* 11.5/100 person-years (95% CI 9.7 to 13.4). An incident STI diagnosis was associated with low alcohol use (adjusted incidence rate ratio (aIRR) 1.3; 95% CI 1.0 to 1.6) and moderate alcohol use (aIRR 1.4; 95% CI 1.1 to 1.8), and having an STI diagnosed at enrolment (aIRR 1.8; 95% CI 1.5 to 2.1).

**Conclusion:**

The high prevalence and incidence of STIs among African women initiating PrEP, most of whom did not report symptoms, highlights the need for aetiologic testing to detect STIs, guide treatment and reduce reproductive health sequelae and risk of transmission.

**Trial registration number:**

clinicaltrials.gov NCT05746065.

**WHAT IS ALREADY KNOWN ON THIS TOPIC**

- HIV pre-exposure prophylaxis (PrEP) programmes in sub-Saharan Africa identify sexually active women who are at risk of curable sexually transmitted infections (STIs), which if not treated are associated with adverse reproductive health consequences. Additional epidemiological data are necessary to understand the prevalence, incidence and risk factors for curable STIs which will guide control measures.

**WHAT THIS STUDY ADDS**

- Young women in Southern and Eastern Africa initiating PrEP had a high prevalence of curable STIs, with 31% having one or more STIs at enrolment, and an incidence rate of 49.3/100 person-years (95% CI 45.3 to 53.4). Approximately 85% of women with prevalent and incident STIs did not report symptoms and would have been missed by syndromic management. Women reporting low or moderate alcohol use at enrolment and diagnosed with an STI at enrolment had an increased risk of diagnosis of an incident STI during follow-up.

**HOW THIS STUDY MIGHT AFFECT RESEARCH, PRACTICE OR POLICY**

- The high prevalence and incidence of curable STIs, most of which were asymptomatic, among young women in Southern and Eastern Africa who were initiating PrEP highlights the need for aetiologic screening to identify asymptomatic infections and guide treatment. Syndromic management is associated with undertreatment due to the high proportion of STIs that are asymptomatic as well as unnecessary antibiotics based on low specificity of vaginal discharge symptoms. PrEP programmes are a priority for STI screening and treatment of young African women, and those with an STI diagnosis should be prioritised for repeat testing and targeted efforts to treat their sexual partners.

## Introduction

 The global burden of curable sexually transmitted infections (STIs) is substantial with 370 million new infections in 2020 among persons aged 15–49 years specifically syphilis (*Treponema pallidum*) infection, *Chlamydia trachomatis*, *Neisseria gonorrhoeae* and *Trichomonas vaginalis*.^1^ Even when asymptomatic, bacterial STIs in women are associated with pelvic inflammatory disease, infertility, ectopic pregnancy, increased risk of HIV acquisition and adverse pregnancy outcomes (eg, early pregnancy loss and congenital syphilis).^2–4^ The mainstay of STI control in low-income and middle-income countries has been condom promotion and syndromic STI management.^5 6^

The scale-up of HIV pre-exposure prophylaxis (PrEP) presents an opportunity to intervene on STIs through diagnosis and treatment, as PrEP services are offered to persons at higher risk for HIV and other STIs, who would benefit from integrated prevention of HIV and other STIs.^7^ In programmes providing PrEP to African women, the prevalence and incidence of any curable STIs (*C. trachomatis*, *N. gonorrhoeae* and *T. vaginalis* combined) have ranged between 30% and 55%.^8–10^ This high STI burden reflects the inadequacy of current STI control efforts and missed opportunities in the delivery of reproductive health services for persons who seek HIV prevention services in low and middle-income countries.^11 12^

Data about the prevalence, incidence and characteristics of STIs among those seeking HIV prevention services could inform HIV and STI control strategies. We assessed the prevalence, incidence and risk factors for curable STIs among African women initiating PrEP in six eastern and southern African countries.

## Methods

### Study design and population

The INSIGHT study was a prospective, open-label cohort study among young women in eastern and southern Africa, which assessed uptake of daily oral PrEP (co-formulated as tenofovir disoproxil fumarate/emtricitabine), adherence and PrEP persistence over 6 months. The study was implemented from August 2022 to July 2023 in six sub-Saharan African countries with 15 sites in South Africa and 1 each in Eswatini, Kenya, Malawi, Uganda and Zambia (Clinicaltrials.gov NCT05746065).

The study population included cisgender women, aged 16–30 years, who were eligible if they were recently sexually active based on reporting vaginal intercourse in the previous 3 months, were interested in PrEP for HIV prevention, and provided written informed consent with parental assent for those aged 16–17 years if required by national guidelines. Participant recruitment occurred through community sensitisation, outreach activities, and referral clinics. Study sites also recruited among women who had indicated interest in research participation in an efficacy trial of islatravir for PrEP, which was suspended earlier that year (IMPOWER 022; clinical trials.gov NCT04644029).

### Study procedures

At the screening visit, data on demographics, HIV testing history, sexual behaviour in the prior 3 months, prior PrEP use, current health status, including acute HIV symptoms, were collected using an interviewer-administered questionnaire. HIV testing was conducted according to national algorithms, and women who screened negative for HIV antibodies and who consented were enrolled into the INSIGHT study, regardless of their decision to initiate PrEP.

At the enrolment visit, study staff administered standardised questionnaires on HIV risk perception, motivations for HIV prevention and PrEP, contraceptive use, substance use including alcohol and recreational drugs, and symptoms of depression and anxiety. Participants were asked about STI symptoms experienced in the 7 days prior to the visit. Participants were counselled about PrEP use and adherence, including safety of use during pregnancy and breastfeeding, and about risk reduction for HIV prevention. Women interested in initiating oral PrEP were offered the option to start daily oral emtricitabine/tenofovir as PrEP at enrolment and start or continue at follow-up visits. At the month 6 visit, participants were provided with a one month refill of PrEP and referred to public PrEP programmes for continuation.

Blood samples were collected at enrolment to diagnose syphilis, and genital swabs were obtained by study staff onsite to test for *C. trachomatis* and *N. gonorrhoeae* and *T. vaginalis*. Participants with symptoms at the enrolment visit were offered same-day STI treatment using syndromic management guidelines, and those without symptoms with positive enrolment laboratory results were provided aetiologic-based treatment the same day if testing was on site, or when the test result was returned to the site, typically within 2 weeks. Appropriate treatment was considered to be doxycycline, erythromycin or azithromycin for *C. trachomatis*; ceftriaxone or cefixime, each alone or with azithromycin for *N. gonorrhoeae*; and metronidazole, tinidazole or secnidazole for *T. vaginalis*, based on national STI treatment guidelines. Participants with reactive syphilis serologies were provided treatment regardless of rapid plasma reagin (RPR) titres, due to lack of historical serological data. Appropriate treatment for syphilis included doxycycline or erythromycin, or intramuscular benzathine penicillin. No test of cure was conducted.

Participants had follow-up visits at months 1, 3 and 6, with HIV rapid testing and STI symptom screening. At months 1 and 3, samples for laboratory STI testing were only collected if symptoms were present. At month 6, all participants had genital swabs collected to test for *C. trachomatis*, *N. gonorrhoeae* and *T. vaginalis*.

## Laboratory methods

HIV testing was conducted using the national rapid testing algorithms for each country. Participants with reactive HIV test results had their samples undergo confirmatory HIV testing with a fourth-generation antigen/antibody test.

For syphilis testing, a RPR test was performed and reactive RPRs had dilutions performed for quantitative titres plus confirmatory testing by *Treponema pallidum* particle agglutination (TPPA). To screen for *C. trachomatis* and *N. gonorrhoeae,* nucleic acid amplification test (NAAT) testing was performed using GenXpert (Cepheid, Sunnyvale, California, USA) on urine or vaginal swabs. *T. vaginalis* was diagnosed by the rapid OSOM test (Seikisui Diagnostics, Burlington, Massachusetts).

To measure recent adherence to PrEP, a point-of-care tenofovir (TFV) lateral flow immunoassay test (Abbott Rapid Diagnostics, Pomona, California, USA) was tested on urine at months 3 and 6, dependent on availability of the urine TFV assay. The assay uses a threshold of >1500 ng/mL for positivity, which indicates TFV ingestion in the prior 4–7 days.^13^

## Statistical analysis

We assessed the baseline prevalence and incidence of aetiologically diagnosed STIs. Prevalence of STIs was defined as having at least one of the four STIs at enrolment including *C. trachomatis*, *N. gonorrhoeae*, *T. vaginalis* and syphilis. We defined having syphilis as a reactive non-treponemal test (e.g., RPR) confirmed by a treponemal-specific test (e.g., TPPA). We estimated the prevalence of participants with potentially active syphilis, defined as a reactive RPR with a titre ≥1:8 and a positive TPPA.

Analyses of incident infection were based on STI results at month 6 when participants were tested for *N. gonorrhoeae*, *C. trachomatis* and *T. vaginalis*. Data for months 1 and 3 visits were excluded as STI testing during these visits was infrequent and only performed based on clinical indications. Syphilis was excluded from incidence calculations as syphilis testing was not performed at month 6. For *C. trachomatis*, *N. gonorrhoeae* and *T. vaginalis*, we defined new infections at month 6 as a positive laboratory result among participants who tested negative at enrolment, or who had received treatment for the same STI at enrolment. Participants with untreated infections at enrolment were excluded from the incidence calculations. Person time at risk was defined as the mid-point time between enrolment and month 6 visit for those with incident STIs, and 6 months for those without incident STIs, given that testing was conducted at two time points.

We used logistic regression to assess baseline demographic or behavioural factors associated with prevalent STIs, including categorical variables for age, contraceptive use, condom use, transactional sex in the past 3 months (sex in exchange for or expectation of receiving material support for self, child or family), intimate partner violence in the past year (physical, emotional or sexual abuse, or feeling unsafe due to a partner’s behaviour), recreational drug use, alcohol use, number of sexual partners, and depressive symptoms based on the Center of Epidemiologic Studies Depression Scale, 10-item version.^14^ Alcohol use in the 3 months prior to enrolment was assessed using the Alcohol Use Disorders Identification Test-Concise (AUDIT-C), a validated 3-item tool (score range 0–12), assessing drinking frequency, quantity and binge drinking.^15^ For the analysis of predictors of prevalent STIs, we used four alcohol use categories based on AUDIT-C scores: non-drinkers (score=0), low-level (score=1–4), medium-level (score=5–8) and high-level (score=9–12).^16^ Variables associated with the outcome at p<0.2 in univariate analysis were included in the multivariable model, and site was included a priori to account for geographic differences in STI prevalence.

To assess factors associated with having an incident STI with either *C. trachomatis*, *N. gonorrhoeae* or *T. vaginalis* infections at month 6, we used Poisson regression to estimate incidence rate ratios (IRRs), with person time included as an offset. We used similar baseline predictor variables as for the prevalence analysis, with the addition of having an enrolment STI. Site was included a priori in our multivariable model, which was adjusted for any predictors with p<0.2 in univariate analysis.

We assessed the association between enrolment STIs and PrEP refills at months 3 and 6, as an indicator of PrEP persistence. Recent PrEP adherence was measured by the urine tenofovir assay. Refill frequency and adherence were summarised by baseline STI status, and associations assessed using logistic regression. We limited the analysis of PrEP adherence by urine tenofovir (TFV) positivity analysis to month 6 only, due to the limited urine test availability at month 3. Both unadjusted and multivariable models were used.

All analyses were conducted using R statistical software V.4.2.3.

## Results

Of the 3087 participants enrolled into the INSIGHT study, 2985 (96.7%) initiated PrEP and 3011 (97.5%) had STI testing results at enrolment. The median age was 23 years (IQR 21–27), 77.2% had secondary education and 96.2% were partnered or married. At enrolment, the majority of women used contraception, approximately a quarter reported engaging in transactional sex within the past 3 months and condom use during last sexual intercourse and one-third reported experiencing intimate partner violence in the past year (table 1). Baseline characteristics differed across countries, with variation in age distribution, contraceptive use, number of sexual partners, intimate partner violence, transactional sex, Vaginal and Oral Interventions to Control the Epidemic (VOICE) risk scores and alcohol use (online supplemental Table 1).

**Table 1:** Baseline sociodemographic and behavioural characteristics of young women from **eastern and southern** Africa participating in the INSIGHT cohort, **2022–2023**

Characteristic	n=3011*	
	**n**	**%, IQR**
**Age (years)**		
Median (IQR)	23	21–27
Age categories in years		
16–21	916	30.4%
22–25	1071	35.6%
25+	1024	34.0%
**Education level**		
No schooling	62	2.1%
Primary	210	6.9%
Secondary	2324	77.2%
Postsecondary education	415	13.8%
**Marital status**		
Single/not partnered	113	3.8%
Partnered/married	2898	96.2%
**Occupation**		
Unemployed or unpaid work in home	1905	63.3%
Student	523	17.4%
Employed with salary/formal	126	4.1%
Employed without a steady salary/informal	457	15.2%
**Contraceptive use**		
No contraceptive use	831	27.6%
Injectable (DMPA, DMPA-SC, NET-EN)	1187	39.4%
Implant	475	15.8%
Male/female condoms only	273	9.1%
Oral contraceptives	154	5.1%
Intrauterine device	36	1.2%
Others†	55	1.8%
**Sexual partners in past 3 months**		
Median (IQR)	1	1–2
**Sexual partners past 3 months**		
≤1	1958	65.0%
2	668	22.2%
3+	385	12.8%
**Condom use during last sex**		
Yes	764	25.4%
No	2247	74.6%
**Intimate partner violence in past 1 year**		
Yes	1103	36.6%
No	1907	63.3%
**Transactional sex in past 3 months**		
Yes	834	27.7%
No	2177	72.3%
**Recreational drug use in past 3 months**		
Yes	248	8.2%
No	2763	91.8%
**Depression symptoms** (CES-D-10 Score)		
Median (IQR)	6	3–10
**Categorical CES-D-10 Scores**		
<10	2246	74.6%
≥10	765	25.4%
**VOICE risk score** ‡		
Median (IQR)	5	4–7
**Alcohol use in past 3 months**		
**Any alcohol use**		
Yes	1966	65.3%
No	1045	34.7%
**Hazardous drinking** (AUDIT-C Score≥3)§		
Yes	1479	49.1%
No	1532	50.9%
**AUDIT-C categories** ^16^		
Non-drinker (score=0)	1045	34.7%
Low (1–4)	989	32.8%
Medium (5–8)	849	28.2%
High risk (9–12)	128	4.3%
**Consumed alcohol with sex in past 1 month**		
Yes	1068	35.5%
No	1943	64.5%

* Data are for 3011 women who had STI testing data from the enrolment visit (i.e., excluded participants missing STI test results data at enrolment).

† Includes intravaginal ring, natural methods, tubal ligation/sterilisation, diaphragm/sponge, other barrier methods, others.

‡ Modified VOICE (Vaginal and Oral Interventions to Control the Epidemic) risk score includes <25 years of age, not being married or cohabiting with a partner, having a primary partner who does not provide financial or material support, having a partner with other sexual partners (reported as yes or maybe), and alcohol use in the past 3 months.

§ Alcohol use was assessed using the AUDIT-C tool: AUDIT-C Score of 3 or more in women is considered indicative of hazardous drinking or active alcohol use disorders.

AUDIT-C, Alcohol Use Disorders Identification Test-Concise; CES-D-10, Center of Epidemiologic Studies Depression Scale, 10-item version; DMPA, Depot Medroxyprogesterone Acetate; DMPA-SC, Depot Medroxyprogesterone Acetate—Subcutaneous; NET-EN, Norethisterone Enanthate.

### Prevalence and incidence of curable STIs

At enrolment, 30.9% of participants had at least one of four curable STIs: 20.8% had *C. trachomatis*, 6.8% *N. gonorrhoeae*, 6.0% *T. vaginalis* and 4.4% had syphilis, with 97 (3.2%) participants having RPR titres ≥ 1:8, indicating possible active syphilis (table 2). Among those with at least one STI, 79.6% had one infection, 17.6% had two STIs and 2.8% had three STIs. Overall, 15.7% of women with a laboratory-confirmed STI reported recent symptoms; 15.5% among those with *C. trachomatis*, 20.9% of those with *N. gonorrhoeae*, 23.1% with *T. vaginalis* and 2.3% of those with syphilis (table 2). Most participants received appropriate treatment for their STIs diagnosed at enrolment: 92.5% of those with *C. trachomatis*, 79.1% among those with *N. gonorrhoeae*, 92.3% of those with *T. vaginalis* and 87.9% of those with syphilis.

**Table 2:** Baseline prevalence and **6 months** incidence of chlamydia, **gonorrhoea**, trichomoniasis and syphilis among women in the INSIGHT cohort

Measure	*Chlamydia trachomatis*	*Neisseria gonorrhoeae*	*Trichomonas vaginalis*	Seropositive* syphilis	≥1 STI diagnosis
**STIs at enrollment** **n=3011**					
**STI Prevalence** n (%)	626 (20.8)	206 (6.8)	182 (6.0)	132 (4.4)	930 (30.9)
**Symptoms among those with a prevalent STI** n (%)	97 (15.5)	43 (20.9)	42 (23.1)	3 (2.3)	146 (15.7)
**Received treatment**	579 (92.5)	163 (79.1)†	168 (92.3)	116 (87.9)	847 (91.1)
**Incident STIs at month 6** (*C. trachomatis*, *N. gonorrhoeae*, *T. vaginalis* only)‡					
**Number of new infections**	374	139	148		574
**Person-years**	1223.5	1281.8	1289.5		1164
**Incidence rate per 100 person years**	30.6	10.8	11.5		49.3
**95% CI**	27.5 to 33.7	9.0 to 12.6	9.7 to 13.4		45.3 to 53.4
**Symptoms among those with an incident STI at month 6** n(%)	51 (13.6)	18 (12.9)	15 (10.1)		73 (12.7)
**Diagnosed with same STI at enrolment** n(%)	115 (30.7)	21 (15.1)	29 (19.6)		127 (27.4)

Proportion with symptoms reflects participants with positive laboratory diagnosis for STI who: reported vaginal discharge for *C. trachomatis*, *N. gonorrhoeae* or *T. vaginalis*.

Reported genital ulcers for syphilis.

* 97 (3.2%) participants had high RPR titres ≥1:8 and a positive confirmatory TPPA, indicative of active syphilis.

† 29/206 (14.1%) participants with *N. gonorrhoeae* were categorized as not receiving appropriate treatment (i.e., received azithromycin only without cefixime or ceftriaxone).

‡ Syphilis is excluded from incidence estimates due to syphilis serologic testing not being performed at month 6.

RPR, rapid plasma reagin; TPPA, *Treponema pallidum* particle agglutination.

At month 6, 2731 (88.5%) participants had laboratory results for *C. trachomatis*, *N. gonorrhoeae* or *T. vaginalis*, and of these, 574 (21.0%) participants had at least one new infection by month 6 (table 2); 86.1% with a single STI, 12.7% with two STIs and 1.2% with three STIs. Overall, 12.7% reported symptoms of those with at least one incident laboratory-confirmed STI at month 6; 13.6% among those with *C. trachomatis*, 12.9% of those with *N. gonorrhoeae* and 10.1% among those with *T. vaginalis* (table 2). Among participants with a new STI, 30.7% with *C. trachomatis*, 15.1% with *N. gonorrhoeae* and 19.6% with *T. vaginalis* had been diagnosed with the same STI at enrolment (table 2). The incidence rate for at least one new STI (either *C. trachomatis*, *N. gonorrhoeae* or *T. vaginalis*) was 49.3 per 100 person-years (95% CI 45.3 to 53.4); 30.6 cases per 100 person-years for *C. trachomatis* (95% CI 27.5 to 33.7), 10.8 cases per 100 person-years for *N. gonorrhoeae* (95% CI 9.0 to 12.6) and 11.5 cases per 100 person-years for *T. vaginalis* (95% CI: 9.7 to 13.4) (table 2).

STI prevalence and incidence rates varied across countries, with the highest burden in Eswatini and South Africa and lower rates in Kenya and Zambia (online supplemental Table 2).

### Correlates of prevalent and incident STIs

Age and any alcohol use in the prior 3 months were significantly associated with having an STI at enrolment: compared with participants 25 years and older, those aged 16–21 (adjusted OR: 1.5; 95% CI 1.2 to 1.9; p*<*0.001) and those aged 22–25 years (adjusted OR: 1.3; 95% CI 1.0 to 1.5; p=0.026) had significantly higher odds of having an STI at enrolment. Participants using hormonal contraception had an increased likelihood of a prevalent STI compared with participants using non-hormonal methods (adjusted OR: 1.3; 95% CI 1.0 to 1.5, p=0.005). Recent alcohol use was associated with having an STI at enrolment: compared with non-drinkers (AUDIT-C score=0), participants with all categories of alcohol use had a higher likelihood of having an STI at enrolment -- low alcohol use (score=1–4) (adjusted OR: 1.3; 95% CI 1.0 to 1.6; p*=*0.019), moderate alcohol use (score=5–8) (adjusted OR: 1.4; 95% CI 1.1 to 1.8; p=0.003) and high alcohol use (score=9–12) (adjusted OR: 1.8; 95% CI 1.2 to 2.7; p=0.006) (table 3).

**Table 3:** Baseline demographic and behavioural factors associated with prevalent and incident STIs among INSIGHT participants

Predictor	Prevalent STI diagnosis at enrolment				Incident STI diagnosis at month 6			
	Unadjusted OR (95% CI)	P value	Adjusted OR (95% CI)	P value	Unadjusted IRR (95% CI)	P value	Adjusted IRR (95% CI)	P value
**Age categories**								
16 to <21	1.5 (1.2 to 1.7)	<0.001	**1.5 (1.2 to 1.9)**	**<0.001**	1.2 (1.0 to 1.4)	0.134	1.1 (0.9 to 1.4)	0.285
22 to 25	1.2 (1.0 to 1.5)	0.042	**1.3 (1.0 to 1.5)**	**0.026**	1.1 (0.9 to 1.3)	0.415	1.0 (0.9 to 1.3)	0.725
25+ (ref)								
**Contraceptive use** *								
Non-hormonal (ref)								
Hormonal	1.3 (1.1 to 1.5)	0.003	**1.3 (1.0 to 1.5)**	**0.005**	1.2 (1.0 to 1.4)	0.053	1.2 (0.9 to 1.4)	0.074
**Number of sexual partners**								
≤1 (ref)								
2	1.1 (0.9 to 1.3)	0.402			1.1 (0.9 to 1.4)	0.171		
3+	0.9 (0.7 to 1.2)	0.456			0.9 (0.7 to 1.2)	0.662		
**Used condom during last vaginal/anal sex**	0.9 (0.8 to 1.1)	0.366			1.0 (0.8 to 1.2)	0.983		
**Experienced intimate partner violence in the past 1 year**	1.0 (0.8 to 1.2)	0.819			1.0 (0.8 to 1.1)	0.613		
**Engaged in transactional sex in the past 3 months**	1.0 (0.8 to 1.2)	0.958			1.0 (0.8 to 1.2)	0.864		
**Used recreational drug in the past 3 months**	1.2 (0.9 to 1.6)	0.136	1.0 (0.8 to 1.4)	0.576	1.2 (0.9 to 1.6)	0.117	1.2 (0.9 to 1.6)	0.241
**Depression symptoms** (CES-D-10 Score)†								
<10 (ref)								
≥10	1.3 (0.6 to 2.8)	0.521			1.0 (0.5 to 2.2)	0.997		
**Alcohol use past 3 months** (AUDIT-C categories)								
Non-drinker (score=0) (ref)								
Low (1–4)	1.5 (1.2 to 1.8)	<0.001	**1.3 (1.0 to 1.6)**	**0.019**	1.4 (1.1 to 1.7)	0.002	**1.3 (1.0 to 1.6**)	**0.050**
Medium (5–8)	1.6 (1.3 to 1.9)	<0.001	**1.4 (1.1 to 1.8)**	**0.003**	1.5 (1.2 to 1.9)	<0.001	**1.4 (1.1 to 1.8)**	**0.009**
High risk (9–12)	1.7 (1.2 to 2.5)	0.005	**1.8 (1.2 to 2.7)**	**0.006**	1.4 (0.9 to 2.1)	0.094	1.2 (0.8 to 1.9)	0.345
**STI at enrolment**					1.9 (1.6 to 2.3)	<0.001	**1.8 (1.5 to 2.1)**	**<0.001**
**Recent PrEP adherence at month 6** Positive urine TFV					0.9 (0.8 to 1.1)	0.261		

Logistic regression was used to assess factors associated with having at least one enrolment STI (*Chlamydia trachomatis*, *Neisseria gonorrhoeae*, *Trichomonas vaginalis* or syphilis) at enrolment. Variables with p<0.2 in univariate analysis were included in the multivariable model, adjusting for site. Poisson regression estimated IRRs for having at least one new infection of either incident *N. gonorrhoeae*, *C. trachomatis* or *T. vaginalis* infections at 6 months.

TFV = tenofovir

Variables which are bolded are statistically significant in the multivariable logistic regression

* Hormonal contraceptives include; injectables (Depot Medroxyprogesterone Acetate, Depot Medroxyprogesterone Acetate—Subcutaneous, Norethisterone Enanthate), implants and oral contraceptives, while non-hormonal/no contraceptives include; intrauterine devices (assumed non-hormonal), condom only, other methods such as natural or other barrier methods, sterilisation/tubal ligation.

† CES-D-10 Score category cut-offs (<10 and ≥10) are from earlier evaluation studies, which show correlation with poor mental health status.

AUDIT-C, Alcohol Use Disorders Identification Test-Concise; CES-D-10, Center of Epidemiologic Studies Depression Scale, 10-item version; IRR, incidence rate ratio; STIs, sexually transmitted infections.

For incident STIs, having a STI at enrolment was significantly associated with having an incident STI at month 6 (adjusted IRR: 1.8; 95% CI 1.5 to 2.1; p<0.001). Alcohol use was significantly associated with having one or more incident STIs; compared with non-drinkers, those with low alcohol use (adjusted IRR: 1.3; 95% CI 1.0 to 1.6; p=0.05), and moderate alcohol use (adjusted IRR: 1.4; 95% CI 1.1 to 1.8; p=0.009) had a higher risk of an incident STI. The other factors assessed, including contraceptive use, depressive symptom scores and adherence to PrEP at month 6, were not associated with having an incident STI (table 3).

### PrEP use association with STIs

PrEP persistence, based on PrEP refills at months 3 or 6, was similar among participants with and without a baseline STI (90.0% vs 89.9%). At month 6, recent PrEP adherence based on urine tenofovir test positivity was similar among participants with and without a baseline STI (62.5% vs 64.0%). In the logistic regression, having an STI at enrolment was not associated with either PrEP refill at month 3 or 6 (adjusted OR: 0.9; 95% CI 0.7 to 1.2; p=0.368) or 6-month urine TFV positivity (adjusted OR: 1.0; 95% CI 0.8 to 1.2; p=0.644) (table 4).

**Table 4:** Association between baseline STI and PrEP refill and **urine tenofovir** positivity at months 3 and 6 in the INSIGHT cohort

Outcome	Timepoint	Had baseline STI n/N(%)		Unadjusted OR (95% CI)	P value	Adjusted OR* (95% CI)	P value
**PrEP refill**	Month 3 or 6	No (ref)	1871/2081 (89.9%)				
		Yes	837/930 (90.0%)	1.0 (0.8 to 1.3)	0.939	0.9 (0.7 to 1.2)	0.368
**Urine TFV positivity** [Table-fn T4_FN2] †	Month 6	No (ref)	972/1519 (64.0%)				
		Yes	430/688 (62.5%)	0.9 (0.8 to 1.1)	0.501	1.0 (0.8 to 1.2)	0.644

* Adjusted for age, any alcohol use, condom use during last sex, number of sexual partners and site.

† Urine tenofovir (TFV) assay when positive indicates recent PrEP use in the prior 4-7 days. Analysis ncludes only those participants who had a urine test at month 6 to detect TFV for measurement of recent PrEP adherence.

PrEP, pre-exposure prophylaxis; STI, sexually transmitted infection.

## Discussion

Among 3087 young women from eastern and southern Africa who were offered daily oral PrEP, 96.5% accepted PrEP, and the 3011 who had STI testing results at enrolment and month 6, the prevalence and incidence of curable STIs were high. At enrolment, one-third of the women had one or more of four curable STIs (*C. trachomatis*, *N. gonorrhoeae*, *T. vaginalis* and syphilis). The incidence of *C. trachomatis*, *N. gonorrhoeae* or TV was almost 50%, with rates varying across the countries. Notably, most curable STIs were asymptomatic; only 15.7% among women with prevalent STIs and 12.7% among those with incident STIs reported symptoms.

Our findings of a high prevalence and incidence of curable STIs are consistent with other studies among women and girls using PrEP in this region. Three quarters of participants were from South Africa, among whom STI rates were higher compared with Eastern African participants, indicating regional differences and a potential need for targeted and context-specific control interventions.^2^ In a prior study in South Africa and Zimbabwe, 55% of women eligible for PrEP had at least one STI at baseline, with incidence rates of 27.8, 11.4 and 6.7 per 100 person-years for *C. trachomatis*, *N. gonorrhoeae* and *T. vaginalis*, respectively.^10^ Another study among women initiating oral PrEP in South Africa reported a baseline prevalence of 25% for *C. trachomatis*, 11% for *N. gonorrhoeae* and 6% for *T. vaginalis*; and an overall STI incidence rate of 52/100 person-year.^8^ In a Kenyan PrEP implementation study among young women, the STI prevalence was 17% for *C. trachomatis* and 8% for *N. gonorrhoeae*, with incidence rates of 40.0 and 12.3 per 100 person-years, respectively.^9 2^

A high proportion of prevalent and incident STIs were asymptomatic, consistent with findings from other studies of STIs among young African women.^8 17 18^ In this study, 85% of young women with prevalent STIs would have been missed by syndromic STI management, which highlights the need for STI diagnostic testing.^6^ In most sub-Saharan African countries, the syndromic approach is the primary method of managing STIs due to its simplicity, ability to provide immediate treatment, ease of integration into primary care and low costs.^1 5 19^ However, syndromic management has limited sensitivity for the detection of asymptomatic STIs and low specificity of the syndromic algorithms among women, which leads to overtreatment and inappropriate antibiotic use, since vaginal discharge symptoms are often non-specific.^19–21^ NAATs are used in high-income countries for the detection of *C. trachomatis*, *N. gonorrhoeae* and *T. vaginalis*, but are currently inaccessible in most African countries due to high costs and limited infrastructure.^22 23^ Low-cost, sensitive STI diagnostic tests are needed to enhance case detection and treatment and prevent reproductive health sequelae, and support STI control interventions, including partner treatment, and support antimicrobial stewardship for preventing development of antimicrobial resistance.^3 24^

In terms of women who could be prioritised for initial STI testing, young women and those who reported alcohol use in the prior 3 months were at increased risk of prevalent and/or incident STIs in our and other studies.^25 26^ Women with an STI diagnosis at enrolment had a higher risk of an incident STI, an indication that they should be prioritised for repeat STI testing and offered treatment for male partners to prevent possible reinfection.^10 25 27^ In our study, partner notification was encouraged, but was not directly facilitated, which may have limited the implementation of partner notification and treatment. We observed an association between hormonal contraceptive use and prevalent but not incident STIs. This finding should be interpreted with caution, given the potential for confounding, and prior studies have not provided conclusive evidence, highlighting the need for further research.^28 29^ Unlike in previous studies, we found no association between self-reported depression scores and having prevalent or incident infections.^10^

We found no significant association between a baseline STI diagnosis and PrEP persistence, as measured by refills at months 3 or 6, or recent PrEP adherence at month 6. Prior studies have shown mixed findings; STI symptoms predicted continued PrEP use at 3 months in Nambia^30^ and a study in California found that baseline STIs were associated with long-term retention on PrEP.^31^ In contrast, a study among men having sex with men found a higher likelihood of PrEP discontinuation among those with an STI.^32^

The study had some limitations including the 6-month follow-up period for ascertainment of incident STIs. Use of the midpoint between enrolment and month 6 for the determination of person-time at risk for incident STIs could have resulted in modest imprecision in person-time calculations; however, we considered this approach since STI testing occurred only at two time points (enrolment and month 6), and participants diagnosed with STIs at enrolment were treated promptly. Information about partner STI treatment was not systematically collected and thus it is difficult to determine the proportion of women with the same STI at enrolment and month 6 who were reinfected from the same partner. We did not conduct a test of cure for *C. trachomatis*, *N. gonorrhoeae* or *T. vaginalis*, which, while not routinely recommended in routine practice except in select circumstances, limits confirmation of microbiological cure and may lead to misclassification of persistent infections as new.^33^ Lastly, the use of the rapid OSOM test for *T. vaginalis* diagnosis, which has a lower sensitivity (75–89%) compared with NAATs, may have led to underestimation of both prevalent and incident *T. vaginalis* infection.^34^

In conclusion, this study highlights the need for diagnostic STI testing in PrEP programmes for young African women who have a high prevalence and incidence of curable STIs, the majority of which are asymptomatic. PrEP programmes provide an opportunity to reach young women who are at risk of adverse outcomes from STIs and would benefit from integrated reproductive health services, including STI testing and treatment and contraceptive services. Increased availability of simple, affordable diagnostic tests in PrEP programmes would enhance STI management and can potentially reduce STI burden in low-income and middle-income countries.

## Supplementary material

10.1136/sextrans-2025-056625 online supplemental file 1

## Data Availability

Data are available on reasonable request.

## References

[1] World Health Organization (2023). Sexually transmitted infections (stis). https://www.who.int/news-room/fact-sheets/detail/sexually-transmitted-infections-(stis).

[2] Jarolimova J, Platt LR, Curtis MR (2022). Curable sexually transmitted infections among women with HIV in sub-Saharan Africa. AIDS.

[3] Vallely LM, Egli-Gany D, Wand H (2021). Adverse pregnancy and neonatal outcomes associated with Neisseria gonorrhoeae: systematic review and meta-analysis. Sex Transm Infect.

[4] Warr AJ, Pintye J, Kinuthia J (2019). Sexually transmitted infections during pregnancy and subsequent risk of stillbirth and infant mortality in Kenya: a prospective study. Sex Transm Infect.

[5] Mason E, Tomlins L, Lewis D (2022). Sexually transmissible infections: current approaches to management. *South African General Practitioner*.

[6] World Health Organization (2024). Updated recommendations for the treatment of neisseria gonorrhoeae, chlamydia trachomatis, and treponema pallidum and new recommendations on syphilis testing and partner services. https://www.who.int/publications/i/item/9789240090767.

[7] Stewart J, Baeten JM (2022). HIV pre-exposure prophylaxis and sexually transmitted infections: intersection and opportunity. Nat Rev Urol.

[8] Gill K, Celum C, Breen G (2019). P432 High prevalence and incidence of curable STIs among young women initiating PrEP in a township in south africa. Sex Transm Infect.

[9] Stewart J, Omollo V, Odoyo J (2019). P424 High prevalence and incidence of bacterial STIs in young women at high risk of HIV prior to PrEP scale-up in kenya. Sex Transm Infect.

[10] Delany-Moretlwe S, Mgodi N, Bekker L-G (2023). High prevalence and incidence of gonorrhoea and chlamydia in young women eligible for HIV pre-exposure prophylaxis in South Africa and Zimbabwe: results from the HPTN 082 trial. Sex Transm Infect.

[11] Ong JJ, Baggaley RC, Wi TE (2019). Global Epidemiologic Characteristics of Sexually Transmitted Infections Among Individuals Using Preexposure Prophylaxis for the Prevention of HIV Infection: A Systematic Review and Meta-analysis. *JAMA Netw Open*.

[12] World Health Organization (2023). Implementation tool for pre-exposure prophylaxis of HIV infection: integrating sti services. https://www.who.int/publications/i/item/9789240057425.

[13] Gati Mirembe B, Donnell D, Krows M (2024). High recent PrEP adherence with point-of-care urine tenofovir testing and adherence counselling among young African women: results from the INSIGHT cohort. J Int AIDS Soc.

[14] Andresen EM, Malmgren JA, Carter WB (1994). Screening for Depression in Well Older Adults: Evaluation of a Short Form of the CES-D. Am J Prev Med.

[15] Bush K, Kivlahan DR, McDonell MB (1998). The AUDIT alcohol consumption questions (AUDIT-C): an effective brief screening test for problem drinking. Ambulatory Care Quality Improvement Project (ACQUIP). Alcohol Use Disorders Identification Test. Arch Intern Med.

[16] Harris AHS, Bradley KA, Bowe T (2010). Associations between AUDIT-C and mortality vary by age and sex. Popul Health Manag.

[17] Korenromp EL, Sudaryo MK, de Vlas SJ (2002). What proportion of episodes of gonorrhoea and chlamydia becomes symptomatic?. Int J STD AIDS.

[18] Mlisana K, Naicker N, Werner L (2012). Symptomatic vaginal discharge is a poor predictor of sexually transmitted infections and genital tract inflammation in high-risk women in South Africa. J Infect Dis.

[19] Gupta V, Sharma VK (2019). Syndromic management of sexually transmitted infections: A critical appraisal and the road ahead. Natl Med J India.

[20] Wi TE, Ndowa FJ, Ferreyra C (2019). Diagnosing sexually transmitted infections in resource-constrained settings: challenges and ways forward. J Int AIDS Soc.

[21] Pettifor A, Walsh J, Wilkins V (2000). How effective is syndromic management of STDs?: A review of current studies. Sex Transm Dis.

[22] Tuddenham S, Hamill MM, Ghanem KG (2022). Diagnosis and Treatment of Sexually Transmitted Infections: A Review. JAMA.

[23] Kenyon C, Herrmann B, Hughes G (2023). Management of asymptomatic sexually transmitted infections in Europe: towards a differentiated, evidence-based approach. *Lancet Reg Health Eur*.

[24] World Health Organization (2023). The diagnostics landscape for sexually transmitted infections. https://www.who.int/publications/i/item/9789240077126.

[25] Chirenje ZM, Gundacker HM, Richardson B (2017). Risk Factors for Incidence of Sexually Transmitted Infections Among Women in a Human Immunodeficiency Virus Chemoprevention Trial: VOICE (MTN-003). Sex Transm Dis.

[26] Kapiga S, Kelly C, Weiss S (2009). Risk factors for incidence of sexually transmitted infections among women in South Africa, Tanzania, and Zambia: results from HPTN 055 study. Sex Transm Dis.

[27] Jongen VW, Schim van der Loeff MF, Botha MH (2021). Incidence and risk factors of C. trachomatis and N. gonorrhoeae among young women from the Western Cape, South Africa: The EVRI study. PLoS One.

[28] Deese J, Pradhan S, Goetz H (2018). Contraceptive use and the risk of sexually transmitted infection: systematic review and current perspectives. *Open Access J Contracept*.

[29] Akter T, Festin M, Dawson A (2022). Hormonal contraceptive use and the risk of sexually transmitted infections: a systematic review and meta-analysis. Sci Rep.

[30] Dzenga T, Moyo E, Moyo P (2023). Factors influencing the retention of clients in oral pre-exposure prophylaxis (PrEP) care at 3 months after initiation in the Omusati region of Namibia. *International Journal of Africa Nursing Sciences*.

[31] Herns S, Panwala R, Pfeil A (2023). Predictors of PrEP retention in at risk patients seen at a HIV primary care clinic in San Diego. Int J STD AIDS.

[32] Hojilla JC, Vlahov D, Crouch PC (2018). HIV Pre-exposure Prophylaxis (PrEP) Uptake and Retention Among Men Who Have Sex with Men in a Community-Based Sexual Health Clinic. AIDS Behav.

[33] Arena CJ, Kenney RM, Eriksson E (2024). Prescribing Practices of Recommended Treatment for Trichomonas vaginalis and Chlamydia trachomatis After 2021 Sexually Transmitted Infection Treatment Guideline Update. Sex Transm Dis.

[34] Gaydos CA, Klausner JD, Pai NP (2017). Rapid and point-of-care tests for the diagnosis of *Trichomonas vaginalis* in women and men. Sex Transm Infect.

