# High prevalence and incidence of curable sexually transmitted infections among young women using oral HIV Pre-exposure prophylaxis in Sub-Saharan Africa; results from the INSIGHT Cohort study

**DOI:** 10.1136/sextrans-2025-056625

**Published:** 2026-08-18

**Authors:** Irene Mukui, Sue Peacock, Deborah Donnell, Brenda Gati Mirembe, Meighan Krows, Elizabeth Bukusi, Loveness Imaan, Philip Kotze, Katherine Gill, Pippa Macdonald, Cheryl Louw, Manjeetha Jaggernath, Anita Marais, Remco P.H. Peters, Sinead Delany-Moretlwe, Amy Ward, Phillip du Preez, Margaret Kasaro, Monica Gandhi, Renee Heffron, Connie Celum

**Affiliations:** 1 Department of Global Health, University of Washington, Seattle, Washington, USA; 2 Fred Hutchinson Cancer Center, Seattle, Washington, USA; 3 Makerere University-Johns Hopkins University Research Collaboration, Kampala, Uganda; 4 Center for Microbiology Research, Kenya Medical Research Institute, Nairobi, Kenya; 5 Johns Hopkins Research Project-Kamuzu University of Health Sciences, Blantyre, Malawi; 6 Qhakaza Mbokodo Research Clinic, Ladysmith, South Africa; 7 Desmond Tutu HIV Centre, Institute of Infectious Disease and Molecular Medicine, Faculty of Health Sciences, University of Cape Town, Cape Town, South Africa; 8 Madibeng Centre for Research, Brits, South Africa; 9 Wits Maternal Adolescent and Child Health Research, Durban, South Africa; 10 Perinatal HIV Research Unit HIV Prevention CRS, Soweto, University of the Witwatersrand, South Africa; 11 Foundation for Professional Development, Research Unit, East London, South Africa; 12 Wits RHI, University of the Witwatersrand, Johannesburg, South Africa; 13 Vuka Research Clinic, Dept of Medicine University of Cape Town, South Africa; 14 University of North Carolina Global Projects Zambia, Lusaka, Zambia; 15 Department of Medicine, University of California, San Francisco, California United States; 16 Department of Medicine, University of Alabama at Birmingham, Birmingham, Alabama, United States; 17 Departments of Medicine and Epidemiology, University of Washington, Seattle, United States

**Keywords:** Sexually transmitted infections, PrEP, young women, Sub-Saharan Africa, incidence, prevalence

## Abstract

**Background:**

HIV pre-exposure prophylaxis (PrEP) programs in Africa reach young women at risk of sexually transmitted infections (STIs). We evaluated curable STI prevalence, incidence, and risk factors among women initiating PrEP.

**Methods:**

From August-December 2022, sexually active women ages 16–30 years from 15 South African sites, and one site each in Eswatini, Kenya, Malawi, Uganda, and Zambia were enrolled into the INSIGHT cohort and offered oral emtricitabine/tenofovir PrEP with follow-up at 1, 3 and 6 months. At each visit, STI symptoms were assessed and treatment provided based on syndromic management or diagnostic testing. Diagnostic tests included nucleic acid amplification for *Chlamydia trachomatis* (CT) and *Neisseria gonorrhoeae* (NG), the rapid OSOM^™^ for *Trichomonas vaginalis* (TV) at enrolment and month 6, and serologic testing for syphilis at enrolment using rapid plasma reagin (RPR) with confirmatory *Treponema pallidum* particle agglutination (TPPA). Prevalence and incidence of each STI were calculated, and predictors assessed using multivariable regression.

**Results:**

Of 3,087 participants, median age was 23 (IQR 21–27), 30.9% had ≥1 STI, with 15.7% reporting symptoms. The prevalence of CT, NG, TV and syphilis was 20.8%, 6.8%, 6.0%, and 4.2%, respectively. The incidence of ≥1 STI (CT, NG, or TV) was 49.3/100 person-years (95%CI: 45.3–53.4) with 12.7% reporting symptoms. The incidence of CT was 30.6/100 person-years (95%CI 27.5–33.7), NG 10.8/100 person-years (95%CI 9.0–12.6), and TV 11.5/100 person-years (95%CI 9.7–13.4). An incident STI diagnosis was associated with low (aIRR 1.3; 95% CI: 1.0–1.6) and moderate (aIRR 1.4; 95% CI: 1.1–1.8) alcohol use, and with having an enrolment STI (aIRR 1.8; 95% CI: 1.5–2.1).

**Conclusion:**

The high prevalence and incidence of STIs among African women initiating PrEP, most of whom did not report symptoms, highlights the need for etiologic testing to detect STIs, guide treatment, and reduce reproductive health sequelae and risk of transmission.

**Clinical trials registration:**

clinicaltrials.gov NCT05746065

## Introduction

The global burden of curable sexually transmitted infections (STIs) is substantial with 370 million new infections in 2020 among persons aged 15–49 years specifically syphilis (*Treponema pallidum* (TP)) infection, *Chlamydia trachomatis* (CT), *Neisseria gonorrhoeae* (NG), and *Trichomonas vaginalis* (TV).^1^ Even when asymptomatic, bacterial STIs in women are associated with pelvic inflammatory disease, infertility, ectopic pregnancy, increased risk of HIV acquisition, and adverse pregnancy outcomes (e.g., early pregnancy loss and congenital syphilis).^2–4^ The mainstay of STI control in low- and middle-income countries has been condom promotion and syndromic STI management.^5,6^

The introduction and scale up of HIV pre-exposure prophylaxis (PrEP) presents an opportunity to intervene on STI acquisition, as PrEP services are offered to persons at higher risk for HIV and other STIs, who would benefit from integrated prevention of HIV and other STIs.^7^ In programs providing PrEP to African women, the prevalence and incidence of any curable STIs (CT, NG, and TV combined) has been 30–55%.^8–10^ The high STI burden reflects the inadequacy of current STI control efforts and missed opportunities in delivery of services for persons who seek HIV prevention services in low and middle income countries.^11,12^

Data about the prevalence, incidence, and characteristics of STIs among those seeking HIV prevention services could inform HIV and STI control strategies. We assessed the prevalence, incidence, and risk factors for curable STIs among African women initiating PrEP in six east and southern African countries.

## Methods

### Study design and population

The INSIGHT study was a prospective, open label cohort study among young women in eastern and southern Africa, which assessed uptake of daily oral tenofovir disoproxil fumarate/emtricitabine (TDF/FTC) PrEP, adherence and PrEP persistence over 6 months. The study was implemented from August 2022 to July 2023 in six sub-Saharan African countries with 15 sites in South Africa and one each in Eswatini, Kenya, Malawi, Uganda, and Zambia (Clinicaltrials.gov NCT05746065).

The study population included cisgender women, ages 16–30 years, who were eligible if they were recently sexually active based on reporting vaginal intercourse in the previous 3 months, were interested in PrEP for HIV prevention, and provided written informed consent with parental assent for those aged 16–17 years if required by national guidelines. Participant recruitment occurred through community sensitization and outreach activities and from referral clinics. Study sites also recruited among women who had indicated interest in research participation following the suspension of an efficacy trial of islatravir (IMPOWER 022; clinical trials.gov NCT04644029).

### Study procedures

At the screening visit, data on demographics, HIV testing history, sexual behaviour in the prior 3 months, prior PrEP use, current health status, including acute HIV and COVID symptoms, were collected using an interviewer-administered questionnaire. HIV testing was conducted according to national algorithms, and women who screened negative for HIV antibodies and consented were enrolled into the INSIGHT study regardless of their decision to initiate PrEP.

At the enrolment visit, study staff administered standardized questionnaires on HIV risk perception, motivations for HIV prevention and PrEP, contraceptive use, substance use including alcohol and recreational drugs, and symptoms of depression and anxiety. Participants were asked about STI symptoms experienced in the seven days prior to the visit. Participants were also counseled about PrEP use and adherence, including use during pregnancy and breastfeeding, and about risk reduction for HIV prevention. Women interested in initiating oral PrEP were offered the option to start or continue daily oral emtricitabine/tenofovir at enrollment, as well as at any follow-up visit. At the month 6 visit, participants were provided with one month PrEP and referred to public PrEP programs for continuation. Blood samples were collected at enrolment to diagnose syphilis, and genital swabs obtained by study staff onsite to test for CT and NG, and TV. Participants with symptoms at the enrolment visit were offered same day treatment using syndromic management guidelines, and those without symptoms with positive enrolment laboratory results were provided etiologic-based treatment same day if testing was onsite, or when the test result was returned to the site typically within two weeks. Appropriate treatment included doxycycline, erythromycin or azithromycin for CT; ceftriaxone or cefixime, each alone or with azithromycin for NG; and metronidazole, tinidazole, or secnidazole for TV, based on national STI treatment guidelines. Participants with reactive syphilis serologies were provided treatment regardless of RPR titers, due to lack of historical serologic data. Appropriate treatment for syphilis included doxycycline or erythromycin, or intramuscular benzathine penicillin. No test of cure was conducted.

Participants had follow-up visits at months 1, 3 and 6, with HIV rapid testing and STI symptom screening. At months 1 and 3, samples for laboratory STI testing were only collected if symptoms were present. At month 6, all participants had genital swabs collected to test for CT, NG, and TV.

### Laboratory methods

HIV testing was conducted using the national rapid testing algorithms for each country. Participants with reactive HIV test results had samples undergo confirmatory HIV testing with a fourth-generation antigen/antibody test.

For syphilis testing, a rapid plasma reagin (RPR) test was performed and those with a reactive RPR had dilutions performed for quantitative titers plus confirmatory testing by *Treponema pallidum* particle agglutination (TPPA). To screen for CT and NG, nucleic acid amplification test (NAAT) testing was performed using GenXpert (Cepheid, Sunnyvale, California) on urine or vaginal swabs. TV was diagnosed by the rapid OSOM test (Seikisui Diagnostics, Burlington, Massachusetts).

To measure adherence to PrEP, a point-of-care urine tenofovir lateral flow immunoassay test (Abbott Rapid Diagnostics, Pomona, CA) was used at month 6. The assay uses a threshold of >1500 ng/ml for positivity which indicates tenofovir ingestion in the prior 4–7 days.^13^

### Statistical analysis

We assessed the baseline prevalence and incidence of etiologically diagnosed STIs. Prevalence of STIs was defined as having at least one of the four STIs at enrolment including CT,NG, TV and syphilis. We defined having syphilis as concurrent positivity on both a non-treponemal test (the rapid plasma reagin-RPR) and a treponemal test (TPPA). We separately estimated the prevalence of participants with potentially active syphilis, defined as a reactive RPR with a titer ≥1:8 and a positive TPPA.

Analyses of incident infection used STI results at month 6 when all participants were tested for NG, CT, and TV. Data for months 1 and 3 visits were excluded as STI testing during these visits was only performed based on clinical indication. Syphilis was excluded from incidence calculation as syphilis testing was not performed at month 6. For CT, NG, and TV, we defined new infections at month 6 as a positive laboratory result among participants who tested negative at enrolment, or who had received treatment for an STI at enrolment. Participants with untreated enrolment infections were excluded from the incidence calculations. Person time at risk was defined as the mid-point time between enrolment and month six visit for those with incident STIs, and 6 months for those without incident STIs, given that testing was conducted at two time points.

We used logistic regression to assess baseline demographic or behavioural factors associated with prevalent STIs: including categorical variables for age, contraceptive use, condom use, transactional sex in the past three months(sex in exchange for or expectation of receiving material support for self, child or family), intimate violence partner in the past year(physical, emotional, or sexual abuse, or feeling unsafe due to a partner's behaviour), recreational drug use, alcohol use, number of sexual partners, and depressive symptoms score based on the self-reported Center of Epidemiologic Studies Depression Scale, 10-item version.^14^ Alcohol use in the three months prior to enrolment was assessed using the AUDIT-C, a validated 3-item tool (score range 0–12), assessing drinking frequency, quantity, and binge drinking.^15^ For the analysis of predictors of prevalent STIs, we used four alcohol use categories based on AUDIT-C scores: non-drinkers (score = 0), low-level (score =1–4), medium-level (score =5–8), and high-level (score =9–12).^16^ Variables associated with the outcome at p<0.2 in univariate analysis were included in the multivariable model, site was included *a priori* to account for geographic differences in STI prevalence.

To assess factors associated with having incident STI with either CT, NG, or TV infections at month 6, we used Poisson regression to estimate incidence rate ratios (IRRs), with person time included as an offset. We used similar baseline predictor variables as for the prevalence analysis, with the addition of having an enrolment STI. Site was included *a priori* in our multivariable model, which was adjusted for any predictors with p<0.2 in univariate analysis.

We assessed the association between enrolment STIs and PrEP refills at months 3 and 6, as an indicator of PrEP persistence, and recent PrEP adherence was measured by urine tenofovir positivity. Refill frequency and adherence were summarized by baseline STI status, and associations assessed using logistic regression. We limited urine tenofovir positivity analysis to month 6 only, due to limited urine test availability at month 3. Both unadjusted and multivariable models were used.

All analyses were conducted using R statistical software version 4.2.3.

### Ethical Approval

The study protocol was approved by ethics review committees for each site in the six study countries. All participants provided written informed consent. For participants below the legal age of consent who were neither emancipated nor mature minors, parental or guardian consent and participant assent was obtained following country specific requirements. The University of Washington Human Subjects Division reviewed the study protocol (IRB Study ID STUDY00015634) and provided a “Not Engaged” determination as per federal regulations.

## Results

Of the 3,087 participants enrolled into the INSIGHT study, 96.7% initiated PrEP, and 97.5% had STI testing results at enrolment. The median age was 23 years (IQR 21–27), 77.2% had secondary education, and 96.2% were partnered or married. At enrolment, majority of women used contraception, approximately one-quarter reported engaging in transactional sex within the past three months and condom use during last sexual intercourse, and one-third reported experiencing intimate partner violence in the past year (Table 1). Baseline characteristics differed across countries, with variation in age distribution, contraceptive use, number of sexual partners, intimate partner violence, transactional sex, VOICE risk scores, and alcohol use (Supplemental Table 1).

### Prevalence and incidence of curable STIs

At enrolment, 30.9% of participants had at least one of four curable STIs: 20.8% had CT, 6.8% NG, 6.0% TV, and 4.4% had syphilis, with 97(3.2%) participants having RPR titers ≥1:8, indicating possible active syphilis (Table 2) . Among those with at least one STI, 79.6% had one infection, 17.6% had two STIs, and 2.8% had three STIs. Overall, 15.7% of women with at least one laboratory-confirmed STI reported recent symptoms; 15.5% among those with CT, 20.9% of those with NG, 23.1% with TV and 2.3% of those with syphilis (Table 2). Most participants received appropriate treatment for their STIs diagnosed at enrolment: 92.5% of those with CT, 79.1% among those with NG, 92.3% of those with TV, and 87.9% of those with syphilis.

At month 6, 2731 (88.5%) participants had laboratory results for CT, NG, or TV, and of these, 574 (21.0%) participants had at least one new infection by month 6(Table 2); 86.1% with a single STI, 12.7% with two STIs, and 1.2% with three STIs. Overall, 12.7% of those with at least one incident laboratory confirmed STI at month 6 reported symptoms; 13.6% among those with CT, 12.9% of those with NG, and 10.1% among those with TV (Table 2). Among participants with a new STI, 30.7% with CT, 15.1% with NG, and 19.6% with TV had been diagnosed with the same STI at enrolment. (Table 2). The incidence rate for at least one new STI (either CT, NG, or TV) was 49.3 per 100 person-years (95% CI 45.3–53.4); 30.6 cases per 100 person-years for CT (95% CI: 27.5–33.7), 10.8 cases per 100 person-years for NG (95% CI: 9.0–12.6), and 11.5 cases per 100 person-years for TV (95% CI: 9.7–13.4), (Table 2).

Both prevalence and incidence rates varied across countries, with the highest burden in eSwatini and South Africa and lower rates in Kenya and Zambia( Supplemental Table 2 ).

### Correlates of prevalent and incident STIs

Age and any alcohol use in the prior 3 months were significantly associated with having an STI at enrolment: compared to participants 25 years and older, those aged 16–21 (adjusted OR: 1.5; 95% CI: 1.2–1.9; *p*<0.001), and those aged 22–25 years (adjusted OR: 1.3; 95% CI: 1.0–1.5; *p*=0.026) had significantly higher odds of having an STI at enrolment. Women using hormonal contraception had increased odds of prevalent STIs compared with those using non-hormonal methods (adjusted OR: 1.3; 95% CI: 1.0–1.5, *p*=0.005). Recent alcohol use was associated with having an STI at enrolment: compared to non-drinkers (AUDIT-C score = 0), participants with low alcohol use (score =1–4) had higher odds of having an enrollment STI (adjusted OR: 1.3; 95% CI: 1.0–1.6; *p*=0.019), as did those with moderate-use (score=5–8) (adjusted OR: 1.4; 95% CI: 1.1–1.8; *p*=0.003) and high use (score = 9–12) (adjusted OR: 1.8; 95% CI: 1.2–2.7; *p*=0.006). (Table 3).

For incident STIs, having a STI at enrolment was significantly associated with having an incident STI at month 6 (adjusted IRR: 1.8; 95% CI: 1.5–2.1; *p*<0.001). Alcohol use was significantly associated with having ≥ one incident STI: compared to non-drinkers, those with low alcohol use (adjusted IRR: 1.3; 95% CI: 1.0–1.6; *p*=0.05), as well as those with moderate alcohol use (adjusted IRR: 1.4; 95% CI: 1.1–1.8; *p*=0.009) had higher risks. The other factors assessed, including contraceptive use, depressive symptom scores, adherence to PrEP at month 6, were not associated with having an incident STI (Table 3).

### PrEP use association with STIs

PrEP persistence, based on PrEP refills at months 3 or 6, was similar among participants with and without a baseline STI (90.0% vs. 89.9%). At month 6, recent PrEP adherence based on tenofovir test positivity, was similar among participants with and without a baseline STI (62.5% vs. 64.0%). Having a STI at enrolment was not associated with either PrEP refill at month 3 or 6 (adjusted OR: 0.9; 95% CI: 0.7–1.2; p=0.368) or 6-month urine TFV positivity (adjusted OR: 1.0; 95% CI: 0.8–1.2; p=0.644) (Table 4).

## Discussion

Among 3087 young women from eastern and southern Africa initiating oral PrEP, the prevalence and incidence of curable STIs was high. At enrolment, one third of the women had at least one of four curable STIs (CT, NG, TV and syphilis), and the incidence of CT, NG, or TV was almost 50%, with rates varying across the countries. Notably, most curable STIs were asymptomatic; only 15.7% among women with prevalent STIs and 12.7% among those with incident STIs reported symptoms.

Our findings of a substantial prevalence and incidence of curable STIs are consistent with other studies among women and girls using PrEP in this region. In a prior study in South Africa and Zimbabwe, 55% of women eligible for PrEP had at least one STI at baseline, with incidence rates of 27.8, 11.4, and 6.7 per 100 person-years for CT, NG, and TV, respectively.^10^ Another study among women initiating oral PrEP in South Africa reported baseline prevalence of 25% for CT, 11% for NG, and 6% for TV; and an overall STI incidence rate of 52/100 person-year.^8^ In Kenya, a PrEP implementation study among young women reported a baseline STI prevalence of 17% for CT and 8% for NG, and incidence rates of 40.0 and 12.3 per 100 person-years, respectively.^9^ Despite the overrepresentation of participants from South Africa, the higher STI rates observed in southern compared to eastern Africa suggest regional differences and the need for targeted and context-specific control interventions.^2^

A high proportion of prevalent and incident STIs were asymptomatic, consistent with findings from other studies of STIs among African women^8,17,18^. In this study, 85% of young women with prevalent STIs would have been missed by syndromic STI management which highlights the need for STI diagnostic testing^6^. In most sub-Saharan African countries, the syndromic approach is the primary method of managing STIs due to its simplicity, ability to provide immediate treatment, ease of integration into primary care, and low costs.^1,5,19^ However, syndromic management is limited for detection and treatment of asymptomatic STIs, and may also lead to overtreatment and inappropriate antibiotic use, as vaginal discharge symptoms are often non-specific for the syndromic management algorithm. ^19–21^ Nucleic acid amplification tests are used in high-income countries for the detection of CT, NG and TV, but are currently inaccessible in many African countries due to high costs and limited infrastructure.^22,23^ Low cost, sensitive STI diagnostic tests are needed to enhance case detection and treatment and prevent reproductive health sequalae, and support STI control interventions, including partner treatment, and support antimicrobial stewardship for preventing development of antimicrobial resistance.^3,24^

In terms of women who could be prioritized for initial STI testing, young women and those who reported alcohol use in the prior 3 months were at increased risk of prevalent and/or incident STIs in our and other studies.^25,26^ Women with an STI diagnosis at enrolment had a higher risk of an incident STI, an indication that they should be prioritized for repeat STI testing, and offered treatment for male partners to prevent possible re-infection.^10,25,27^ In our study, partner notification was voluntary, which may have limited the implementation of partner notification and treatment. We observed an association between hormonal contraceptive use and prevalent but not incident STIs. This finding should be interpreted with caution given the potential for confounding, and prior studies have not provided conclusive evidence, highlighting the need for further research.^28,29^ Unlike in previous studies, we found no association between self-reported depression scores and having prevalent or incident infections.^10^

We found no significant association between a baseline STI diagnosis and PrEP persistence, as measured by refills at months 3 or 6, or PrEP adherence at month 6. Prior studies have shown mixed findings: in Namibia, STI symptoms predicted continued PrEP use at three months^30^; and in San Diego baseline STIs were associated with long term retention on PrEP.^31^ In contrast, a study among men having sex with men found a higher likelihood of PrEP discontinuation among participants with an STI.^32^

The study had some limitations including the 6-month follow-up period for ascertainment of incident STIs. Use of the midpoint between enrolment and month 6 for the determination of person-time at risk for incident STIs could have introduced some imprecision in person time calculations, however we considered this approach since STI testing occurred only at two time points (enrollment and month 6), and participants diagnosed with STIs at enrollment were treated promptly. Information about partner STI treatment was not systematically collected and thus it is difficult to determine the proportion of women with the same STI at enrolment and month 6 who were reinfected from the same partner. We did not conduct a test of cure for CT, NG, or TV, which while not routinely recommended in routine practice except in select circumstances, limits confirmation of microbiological cure and may lead to misclassification of persistent infections as new.^33^ Lastly, the use of the rapid OSOM^™^ test for TV diagnosis, which has a lower sensitivity (75–89%) compared to NAATs, may have led to underestimation of both prevalent and incident TV infection.^34^

In conclusion, this study highlights the need for diagnostic STI testing among PrEP programs for young African women who have a high prevalence and incidence of curable STIs, the majority of which are asymptomatic. PrEP programs provide an opportunity to reach young women who are at risk of adverse outcomes from STIs and would benefit from integrated reproductive health services, including STI testing and treatment and contraceptive services. Increased availability of simple, affordable diagnostic tests in PrEP programs would enhance STI management and can potentially reduce STI burden in low- and middle-income countries.

## Supplementary Material

Supp1

## Figures and Tables

**Table 1:** Baseline sociodemographic and behavioural characteristics of young women from East and Southern Africa participating in the INSIGHT cohort, 2022–2023

Characteristic	N = 3011*	
	N	%, IQR

**Age in years**		
Median (IQR)	23	21–27
**Age categories in years**		
16–21	916	30.4%
22–25	1071	35.6%
25+	1024	34.0%
**Education level**		
No schooling	62	2.1%
Primary	210	6.9%
Secondary	2324	77.2%
Post-secondary education	415	13.8%
**Marital status**		
Single/not partnered	113	3.8%
Partnered/married	2898	96.2%
**Occupation**		
Unemployed/housewife	1905	63.3%
Student	523	17.4%
Employed with salary/formal	126	4.1%
Employed without a steady salary/informal	457	15.2%
**Contraceptive use**		
No contraceptive use	831	27.6%
Injectable (DMPA, DMPA-SC, NET-EN)	1187	39.4%
Implant	475	15.8%
Male/Female condoms only	273	9.1%
Oral contraceptives	154	5.1%
Intra-uterine device	36	1.2%
Others**	55	1.8%
**Number of sexual partners in past 3 months**		
Median (IQR)	1	1–2
**Number of sexual partners past 3 months (categories)**		
≤1	1958	65.0%
2	668	22.2%
3+	385	12.8%
**Condom use during last sex**		
Yes	764	25.4%
No	2247	74.6%
**Intimate partner violence in past 1 year**		
Yes	1103	36.6%
No	1907	63.3%
**Transactional sex in past 3 months**		
Yes	834	27.7%
No	2177	72.3%
**Recreational drug use in past 3 months**		
Yes	248	8.2%
No	2763	91.8%
**CES-D-10 score**		
Median (IQR)	6	3–10
**CES-D-10 score (categories)**		
<10	2246	74.6%
≥10	765	25.4%
**VOICE risk score** ^a^		
Median (IQR)	5	4–7
**Alcohol use in past 3 months Any use in past 3 months**		
Yes	1966	65.3%
No	1045	34.7%
**Hazardous drinking (AUDIT-C score ≥3)** ^b^		
Yes	1479	49.1%
No	1532	50.9%
**AUDIT-C Categories** ^16^		
Non-drinker (score=0)	1045	34.7%
Low ( 1–4)	989	32.8%
Medium(5–8)	849	28.2%
High risk (9–12)	128	4.3%
**Had alcohol with sex in past 1 month**		
Yes	1068	35.5%
No	1943	64.5%

* Data is for 3011 women who had STI testing data from the enrolment visit (excluded participants are those missing testing results data at enrolment)

** Includes intravaginal ring, natural methods, tubal ligation/sterilization, diaphragm/sponge, other barrier methods, others

a Modified VOICE risk score; included <25 years of age, not being married or cohabiting with a partner, having a primary partner who does not provide financial or material support, having a partner with other sexual partners (reported as yes or maybe), and alcohol use in the past three months.

b Alcohol use was assessed using the AUDIT-C tool:
AUDIT-C score of 3 or more in women is considered positive for hazardous drinking or active alcohol use disorders

**Abbreviations**: DMPA-Depot Medroxyprogesterone Acetate; DMPA-SC- Depot Medroxyprogesterone Acetate – Subcutaneous; NET-EN- Norethisterone Enanthate; AUDIT-C-Alcohol Use Disorders Identification Test-Concise; CES-D-10 - Center of Epidemiologic Studies Depression Scale, 10-item version

**Table 2:** Baseline prevalence and six months incidence of chlamydia, gonorrhea, trichomoniasis, and syphilis among women in the INSIGHT cohort

Measure	*Chlamydia trachomatis*	*Neisseria gonorrhoeae*	*Trichomonas vaginalis*	Sero-positive* Syphilis	Have ≥ 1 STI
**Baseline Prevalence N=3011**					
**Prevalence n(%)**	626 (20.8)	206 (6.8)	182 (6.0)	132 (4.4)	930 (30.9)
Symptomatic n(%)	97 (15.5)	43 (20.9)	42 (23.1)	3 (2.3)	146 (15.7)
Received treatment	579 (92.5)	163 (79.1)**	168 (92.3)	116 (87.9)	847 (91.1)
**Incidence at month 6 CT, NG, TV only)** ***					
Number of new infections	374	139	148		574
Person-years	1223.5	1281.8	1289.5		1164
Incidence rate per 100 person years	30.6	10.8	11.5		49.3
95% Confidence Interval	27.5–33.7	9.0 – 12.6	9.7–13.4		45.3 – 53.4
Symptomatic at month 6 n(%)	51 (13.6)	18 (12.9)	15 (10.1)		73 (12.7)
Had same STI at enrolment n(%)	115 (30.7)	21 (15.1)	29 (19.6)		127 (27.4)

Proportion with symptoms reflects participants with positive laboratory diagnosis for STI who:
Reported vaginal discharge for CT, NG, or TV Reported genital ulcers for syphilis

* 97 (3.2%) of participants had high RPR titres ≥ 1:8 and positive TPPA , suggestive of active syphilis

** 29/206 (14.1%) of participants with NG were excluded from appropriate treatment as they received azithromycin only ( i.e. without cefixime or ceftriaxone)

*** Syphilis is excluded from incidence estimates due no testing at month 6

**Abbreviations**: CT = Chlamydia trachomatis, NG = Neisseria gonorrhoeae, TV = Trichomonas vaginalis

**Table 3:** Baseline demographic and behavioural factors associated with prevalent and incident STIs among INSIGHT participants

	Prevalent STI diagnosis at enrollment				Incident STI diagnosis at month 6			
Predictor	Unadjusted OR (95% CI)	p-value	Adjusted OR (95% CI)	p-value	Unadjusted IRR (95% CI)	p-value	Adjusted IRR (95% CI)	p-value

**Age Categories**								
Age 16-<21	1.5 (1.2 −1.7)	<0.001	**1.5 (1.2–1.9)**	**<0.001**	1.2 (1.0 −1.4)	0.134	1.1 ( 0.9–1.4)	0.285
Age 22–25 Age 25+(ref)	1.2 (1.0 −1.5)	0.042	**1.3 (1.0–1.5)**	**0.026**	1.1 (0.9 −1.3)	0.415	1.0 (0.9–1.3)	0.725
**Contraceptives** *								
Non-hormonal (ref) Hormonal	1.3(1.1 – 1.5)	0.003	**1.3(1.0 – 1.5)**	**0.005**	1.2(1.0 – 1.4)	0.053	1.2(0.9–1.4)	0.074
**Number of sexual partners**								
≤1 (ref)								
2	1.1(0.9 – 1.3)	0.402			1.1( 0.9 – 1.4)	0.171		
3+	0.9(0.7 – 1.2)	0.456			0.9(0.7 – 1.2)	0.662		
**Used condom during last vaginal/anal sex**	0.9 (0.8 –1.1)	0.366			1.0 (0.8 – 1.2)	0.983		
**Experienced intimate partner violence past 1 year**	1.0 (0.8 −1.2)	0.819			1.0 (0.8 – 1.1)	0.613		
**Engaged in transactional sex in the past 3 months**	1.0 (0.8 −1.2)	0.958			1.0 (0.8 – 1.2)	0.864		
**Used recreational drug in the past 3 months**	1.2 (0.9 −1.6)	0.136	1.0 (0.8– 1.4)	0.576	1.2 (0.9 – 1.6)	0.117	1.2 (0.9–1.6)	0.241
**CES-D-10 score** **								
<10 (ref)								
≥10	1.3 (0.6 – 2.8)	0.521			1.0 (0.5 – 2.2)	0.997		
**Alcohol use past three months (AUDIT-C categories)**								
Non-drinker (score=0) (ref) Low ( 1–4)	1.5 (1.2 −1.8)	<0.001	**1.3 (1.0 – 1.6)**	**0.019**	1.4 (1.1 – 1.7)	0.002	**1.3 (1.0– 1.6)**	**0.050**
Medium(5–8)	1.6 (1.3 −1.9)	<0.001	**1.4 (1.1 – 1.8)**	**0.003**	1.5 (1.2 – 1.9)	<0.001	**1.4 (1.1– 1.8)**	**0.009**
High risk (9–12)	1.7 (1.2–2.5)	0.005	**1.8 (1.2 – 2.7)**	**0.006**	1.4 (0.9 – 2.1)	0.094	1.2 (0.8–1.9)	0.345
**Had an enrollment STI**					1.9 (1.6 – 2.3)	<0.001	**1.8 (1.5–2.1)**	**<0.001**
**Urine TFV positive at month 6**					0.9 (0.8 – 1.1)	0.261		

Logistic regression was used to assess factors associated with having at least one enrolment STI (CT,NG , TV or syphilis) at enrolment. Variables with P < 0.2 in univariate analysis were included in the multivariable model, adjusting for site. Poisson regression estimated incidence rate ratios (IRRs) for having at least one new infection of either incident NG, CT, or TV infections at 6 months.

* Hormonal contraceptives include; injectables (DMPA, DMPA-SC,NET-EN), implants, and oral contraceptives, while non-hormonal/no contraceptives include; intrauterine devices (assumed non-hormonal), condom only, other methods such as natural or other barrier methods, sterilization/ tubal ligation

** CES-D-10 score category cut-offs (<10 & ≥10) are from earlier evaluation studies, that show correlation with poor mental health status ^14^

**Abbreviations**: CES-D-10: Center of Epidemiologic Studies Depression Scale, 10-item version; AUDIT-C-Alcohol Use Disorders Identification Test-Concise

**Table 4:** Association between baseline STI and PrEP refill and Urine Tenofovir positivity at months 3 and 6 in the INSIGHT cohort

Outcome	Timepoint		Had baseline STI n/N(%)	Unadjusted OR (95% CI)	p-value	Adjusted OR* (95% CI)	p-value

**PrEP Refill**	Month 3 or 6	No (ref)	1871 / 2081 (89.9%)				
		Yes	837 / 930 (90.0%)	1.0 (0.8–1.3)	0.939	0.9 (0.7–1.2)	0.368
**TFV Positivity** **	Month 6	No (ref)	972 / 1519 (64.0%)				
		Yes	430 / 688 (62.5%)	0.9 (0.8–1.1)	0.501	1.0 (0.8–1.2)	0.644

* Adjusted for age , any alcohol use , condom use during last sex, number of sexual partners and site

** Includes only those participants who had a urine test at month 6 to detect TFV for measurement of recent PrEP adherence

## Data Availability

The data that support the findings of this study are available from the corresponding author upon reasonable request through icrc@uw.edu, which will be reviewed by a central publications and manuscript committee.

## What is already known about this topic

HIV PrEP programs in sub-Saharan Africa identify sexually active women who are at risk of curable sexually transmitted infections (STIs), which if not treated are associated with adverse reproductive health consequences. Additional epidemiologic data is necessary to understand the prevalence, incidence, and risk factors for curable STIs which will guide control measures.

## What this study adds

Young women in sub-Saharan Africa initiating PrEP had a high prevalence of curable STIs with 31% having one or more STIs at enrolment, and an incidence rate of 49.3/100 person-years (95% CI 45.3–53.4). Approximately 85% of women with prevalent and incident STIs did not report symptoms and would have been missed by syndromic management. Women reporting alcohol use at enrolment and with an enrolment STI had an increased risk of having an incident STI during follow-up.

## How this study might affect research, practice and policy

The high prevalence and incidence of curable STIs, most of which were asymptomatic, among young women in sub-Saharan Africa initiating PrEP highlights the need for etiologic diagnosis and guided treatment. Syndromic management is associated with undertreatment as well as unnecessary antibiotics based on lack of specificity. PrEP programs are a priority for STI screening and treatment, and those with an STI diagnosis should be prioritized for repeat testing and targeted efforts to treat their sexual partners.

## Study sites:

International AIDS Care and Treatment Program, ICAP Eswatini Research Centre (Mbababane, Eswatini), Kenya Medical Research Institute, KEMRI (Kisumu, Kenya), Kamuzu Research Centre (Blantrye, Malawi), Setshaba (Soshanguve, South Africa), Qhakaza Mbokodo Research Centre (Ladysmith, South Africa), Masiphumulele (CapeTown, South Africa), Madibeng Centre for Research (Brits, South Africa), Helen Joseph Clinical HIV Research Unit, CHRU (Johannesburg, South Africa), Emavundleni Research Centre (CapeTown, South Africa), Aurum Rustenburg Clinical Research Centre (Rustenburg, South Africa), Perinatal HIV Research Unit-HIV Prevention Clinical Research Site, PHRU (Soweto, South Africa), Human Sciences Research Council, Centre for Community Based Research, CCBR, Pietermaritzburg South Africa), MATCH Research Unit (Durban, South Africa), Foundation for Professional Development Ndevana Community Research Site (East London, South Africa), Wits Reproductive Health & HIV Research Unit Ward 21 Clinical Research Site (Johannesburg, South Africa), UCT Khayelisha Clinical Research Site (CapeTown, South Africa ), Aurum Klerksdorp Clinical Research Centre (Klerksdorp, South Africa), SA Medical Research Council - Chatsworth Clinical Research Site (Chatsworth, South Africa), Makerere University/Johns Hopkins University (MU-JHU) (Kampala, Uganda) and Kamwala Health Centre (Lusaka, Zambia). We appreciate the commitment and contributions of the INSIGHT site staff.

## References

[1] World Health Organization. Sexually transmitted infections (STIs) [Internet]. Geneva: World Health Organization; [cited 2023 Feb 1]. Available from: https://www.who.int/news-room/fact-sheets/detail/sexually-transmitted-infections-(stis)

[2] Jarolimova J, Platt LR, Curtis MR, Philpotts LL, Bekker LG, Morroni C, Curable sexually transmitted infections among women with HIV in sub-Saharan Africa. AIDS Lond Engl. 2022 Apr 1;36(5):697–709. 10.1097/QAD.0000000000003163 PMC8957553 34999605

[3] Vallely LM, Egli-Gany D, Wand H, Pomat WS, Homer CSE, Guy R, Adverse pregnancy and neonatal outcomes associated with Neisseria gonorrhoeae: systematic review and meta-analysis. Sex Transm Infect. 2021 Mar;97(2):104–11. 10.1136/sextrans-2020-054653 PMC7892372 33436505

[4] Warr AJ, Pintye J, Kinuthia J, Drake AL, Unger JA, McClelland RS, Sexually transmitted infections during pregnancy and subsequent risk of stillbirth and infant mortality in Kenya: a prospective study. Sex Transm Infect. 2019 Feb;95(1):60–6. 10.1136/sextrans-2018-053597 PMC6525108 30228109

[5] Mason E, Tomlins L, Lewis D. Sexually transmissible infections: current approaches to management. South Afr Gen Pract. 2022 Jun;3(3):89–94.

[6] World Health Organization. Updated recommendations for the treatment of Neisseria gonorrhoeae, Chlamydia trachomatis, and Treponema pallidum and new recommendations on syphilis testing and partner services [Internet]. Geneva: World Health Organization; 2024 [cited 2025 Jan 25]. Available from: https://www.who.int/publications/i/item/9789240090767 39137269

[7] Stewart J, Baeten JM. HIV pre-exposure prophylaxis and sexually transmitted infections: intersection and opportunity. Nat Rev Urol. 2022 Jan;19(1):7–15. 10.1038/s41585-021-00527-4 PMC9249100 34697493

[8] Gill K, Celum C, Breen G, Thomas K, Morton J, Baeten J, P432 High prevalence and incidence of curable STIs among young women initiating PrEP in a township in south africa. Sex Transm Infect. 2019 Jul 1;95(Suppl 1):A205–A205.

[9] Stewart J, Omollo V, Odoyo J, Kidoguchi L, Morton J, Johnson R, P424 High prevalence and incidence of bacterial STIs in young women at high risk of HIV prior to PrEP scale-up in kenya. Sex Transm Infect. 2019 Jul 1;95(Suppl 1):A202–A202.

[10] Delany-Moretlwe S, Mgodi N, Bekker LG, Baeten JM, Li C, Donnell D, High prevalence and incidence of gonorrhoea and chlamydia in young women eligible for HIV pre-exposure prophylaxis in South Africa and Zimbabwe: results from the HPTN 082 trial. Sex Transm Infect [Internet]. 2023 Mar 8 [cited 2023 Oct 2]; Available from: https://sti.bmj.com/content/early/2023/03/07/sextrans-2022-055696 10.1136/sextrans-2022-055696 PMC10555488 36889914

[11] Ong JJ, Baggaley RC, Wi TE, Tucker JD, Fu H, Smith MK, Global Epidemiologic Characteristics of Sexually Transmitted Infections Among Individuals Using Preexposure Prophylaxis for the Prevention of HIV Infection: A Systematic Review and Meta-analysis. JAMA Netw Open. 2019 Dec 2;2(12):e1917134. 10.1001/jamanetworkopen.2019.17134 PMC6991203 31825501

[12] World Health Organization. Implementation tool for pre-exposure prophylaxis of HIV infection: integrating STI services [Internet]. Geneva: World Health Organization; 2023 [cited 2024 Aug 20]. Available from: https://www.who.int/publications/i/item/9789240057425

[13] Gati Mirembe B, Donnell D, Krows M, Zwane Z, Bukusi E, Panchia R, High recent PrEP adherence with point-of-care urine tenofovir testing and adherence counselling among young African women: results from the INSIGHT cohort. J Int AIDS Soc. 2024 Dec 9;27(12):e26389. 10.1002/jia2.26389 PMC11628190 39653676

[14] Andresen EM, Malmgren JA, Carter WB, Patrick DL. Screening for Depression in Well Older Adults: Evaluation of a Short Form of the CES-D. Am J Prev Med. 1994 Mar 1;10(2):77–84. 8037935

[15] Bush K, Kivlahan DR, McDonell MB, Fihn SD, Bradley KA, for the Ambulatory Care Quality Improvement Project (ACQUIP). The AUDIT Alcohol Consumption Questions (AUDIT-C): An Effective Brief Screening Test for Problem Drinking. Arch Intern Med. 1998 Sep 14;158(16):1789–95. 10.1001/archinte.158.16.1789 9738608

[16] Harris AHS, Bradley KA, Bowe T, Henderson P, Moos R. Associations Between AUDIT-C and Mortality Vary by Age and Sex. Popul Health Manag. 2010 Oct;13(5):263–8. 10.1089/pop.2009.0060 PMC3135896 20879907

[17] Korenromp EL, Sudaryo MK, de Vlas SJ, Gray RH, Sewankambo NK, Serwadda D, What proportion of episodes of gonorrhoea and chlamydia becomes symptomatic? Int J STD AIDS. 2002 Feb;13(2):91–101. 10.1258/0956462021924712 11839163

[18] Mlisana K, Naicker N, Werner L, Roberts L, van Loggerenberg F, Baxter C, Symptomatic Vaginal Discharge Is a Poor Predictor of Sexually Transmitted Infections and Genital Tract Inflammation in High-Risk Women in South Africa. J Infect Dis. 2012 Jul 1;206(1):6–14. 10.1093/infdis/jis298 PMC3490689 22517910

[19] Gupta V, Sharma VK. Syndromic management of sexually transmitted infections: A critical appraisal and the road ahead. Natl Med J India. 2019 May 1;32:147. 10.4103/0970-258X.278691 32129308

[20] Wi TE, Ndowa FJ, Ferreyra C, Kelly-Cirino C, Taylor MM, Toskin I, Diagnosing sexually transmitted infections in resource-constrained settings: challenges and ways forward. J Int AIDS Soc. 2019 Aug 30;22(Suppl Suppl 6):e25343. 10.1002/jia2.25343 PMC6715950 31468679

[21] Pettifor A, Walsh J, Wilkins V, Raghunathan P. How Effective Is Syndromic Management of STDs?: A Review of Current Studies. Sex Transm Dis. 2000 Aug;27(7):371. 10.1097/00007435-200008000-00002 10949428

[22] Tuddenham S, Hamill MM, Ghanem KG. Diagnosis and Treatment of Sexually Transmitted Infections: A Review. JAMA. 2022 Jan 11;327(2):161–72. 10.1001/jama.2021.23487 35015033

[23] Kenyon C, Herrmann B, Hughes G, de Vries HJC. Management of asymptomatic sexually transmitted infections in Europe: towards a differentiated, evidence-based approach. Lancet Reg Health - Eur. 2023 Oct 26;34:100743. 10.1016/j.lanepe.2023.100743 PMC10624996 37927435

[24] World Health Organization. The diagnostics landscape for sexually transmitted infections [Internet]. Geneva: World Health Organization; 2023 [cited 2025 May 2]. Available from: https://www.who.int/publications/i/item/9789240077126

[25] Chirenje ZM, Gundacker HM, Richardson B, Rabe L, Gaffoor Z, Nair GL, Risk Factors for Incidence of Sexually Transmitted Infections Among Women in a Human Immunodeficiency Virus Chemoprevention Trial: VOICE (MTN-003). Sex Transm Dis. 2017 Mar;44(3):135–40. 10.1097/OLQ.0000000000000568 PMC5535309 28178109

[26] Kapiga S, Kelly C, Weiss S, Daley T, Peterson L, Leburg C, Risk Factors for Incidence of Sexually Transmitted Infections Among Women in South Africa, Tanzania, and Zambia: Results From HPTN 055 Study. Sex Transm Dis. 2009 Apr;36(4):199. 10.1097/OLQ.0b013e318191ba01 19265734

[27] Jongen VW, van der Loeff MFS, Botha MH, Sudenga SL, Abrahamsen ME, Giuliano AR. Incidence and risk factors of C. trachomatis and N. gonorrhoeae among young women from the Western Cape, South Africa: The EVRI study. PLOS ONE. 2021 May 3;16(5):e0250871. 10.1371/journal.pone.0250871 PMC8092667 33939747

[28] Deese J, Pradhan S, Goetz H, Morrison C. Contraceptive use and the risk of sexually transmitted infection: systematic review and current perspectives. Open Access J Contracept. 2018 Nov 12;9:91–112. 10.2147/OAJC.S135439 PMC6239113 30519127

[29] Akter T, Festin M, Dawson A. Hormonal contraceptive use and the risk of sexually transmitted infections: a systematic review and meta-analysis. Sci Rep. 2022 Nov 25;12:20325. 10.1038/s41598-022-24601-y PMC9700818 36434126

[30] Dzenga T, Moyo E, Moyo P, Kamangu J, Dzinamarira T. Factors influencing the retention of clients in oral pre-exposure prophylaxis (PrEP) care at 3 months after initiation in the Omusati region of Namibia. Int J Afr Nurs Sci. 2023 Jan 1;19:100623.

[31] Herns S, Panwala R, Pfeil A, Sardinha M, Rossi V, Blumenthal J, Predictors of PrEP retention in at risk patients seen at a HIV primary care clinic in San Diego. Int J STD AIDS. 2023 Oct 1;34(11):785–90. 10.1177/09564624231179276 PMC10561521 37271811

[32] Hojilla JC, Vlahov D, Crouch PC, Dawson-Rose C, Freeborn K, Carrico A. HIV Pre-exposure Prophylaxis (PrEP) Uptake and Retention Among Men Who Have Sex with Men in a Community-Based Sexual Health Clinic. AIDS Behav. 2018 Apr;22(4):1096–9. 10.1007/s10461-017-2009-x PMC5879003 29243109

[33] Arena CJ, Kenney RM, Eriksson E, Brar I, Veve MP. Prescribing Practices of Recommended Treatment for Trichomonas vaginalis and Chlamydia trachomatis After 2021 Sexually Transmitted Infection Treatment Guideline Update. Sex Transm Dis. 2024 Nov;51(11):e40. 10.1097/OLQ.0000000000001987 38709026

[34] Gaydos CA, Klausner JD, Pai NP, Kelly H, Coltart C, Peeling RW. Rapid and point-of-care tests for the diagnosis of Trichomonas vaginalis in women and men. Sex Transm Infect. 2017 Dec 1;93(S4):S31–5. 10.1136/sextrans-2016-053063 PMC5723541 28684611

