# Supplementary material for: High prevalence and incidence of curable sexually transmitted infections among young women using oral HIV Pre-exposure prophylaxis in Sub-Saharan Africa; results from the INSIGHT Cohort study
Source: Sex Transm Infect. Author manuscript; Available in PMC 2026 Oct 17. (PMC13539827; doi:10.1136/sextrans-2025-056625)
Supplement: Supplementary file 1 [file NIHMS2144697-supplement-Supp1.docx]

**Supplemental material**

**Supplementary table 1: Baseline demographic and behavioural characteristics among INSIGHT participants by country**

| **Variable** | **eSwatini**  **N=146** | **Kenya**  **N=150** | **Malawi**  **N=148** | **South Africa**  **N=2277** | **Uganda**  **N=148** | **Zambia**  **N=142** |
| --- | --- | --- | --- | --- | --- | --- |
| **Age**  **Median (IQR)** | 24 [21–27] | 25 [23–28] | 23 [20–25] | 23 [21–27] | 21.5 [19–25] | 24 [21–27] |
| **Age categories** |  |  |  |  |  |  |
| 16–21 | 43 (29.5%) | 23 (15.3%) | 54 (36.5%) | 683 (30.0%) | 74 (50.0%) | 39 (27.5%) |
| 22–25 | 45 (30.8%) | 55 (36.7%) | 60 (40.5%) | 823 (36.1%) | 39 (26.4%) | 49 (34.5%) |
| 25+ | 58 (39.7%) | 72 (48.0%) | 34 (23.0%) | 771 (33.9%) | 35 (23.6%) | 54 (38.0%) |
| **Marital status** |  |  |  |  |  |  |
| Single/no partner | 6 (4.1%) | 6 (4.0%) | 2 (1.4%) | 56 (2.5%) | 42 (28.4%) | 1 (0.7%) |
| Married/partnered | 140 (95.9%) | 144 (96.0%) | 146 (98.6%) | 2221 (97.5%) | 106 (71.6%) | 141 (99.3%) |
| **Contraceptive use** |  |  |  |  |  |  |
| No contraceptive | 103 (70.5%) | 48 (32.0%) | 40 (27.0%) | 551 (24.2%) | 48 (32.4%) | 41 (28.9%) |
| Injectable | 14 (9.6%) | 33 (22.0%) | 49 (33.1%) | 1016 (44.6%) | 28 (18.9%) | 47 (33.1%) |
| Implant | 18 (12.3%) | 40 (26.7%) | 25 (16.9%) | 341 (15.0%) | 24 (16.2%) | 27 (19.0%) |
| Oral pills | 4(2.7%) | 11(7.3%) | 5(3.4%) | 116(5.1%) | 12(8.1%) | 6(4.2%) |
| Intra-uterine device | 1(0.7%) | 4(2.7%) | 1(0.7%) | 28(1.2%) | 1(0.7%) | 1(0.7%) |
| Male/female condoms only | 5 (3.4) | 8 (5.3%) | 14 (9.5%) | 197 (8.7%) | 32 (21.6%) | 17 (12.0%) |
| Other | 1 (0.7%) | 6(4.0%) | 14(9.5%) | 28 (1.2%) | 3(2.0%) | 3 (2.1%) |
| **Sexual partners (past 3 months) — Median (IQR)** | 1 [1–1] | 2 [1–3] | 1 [1–1] | 1 [1–2] | 3 [2–4] | 1 [1–1] |
| **Number of sexual partners (categories)** |  |  |  |  |  |  |
| ≤1 | 124 (84.9%) | 65 (43.3%) | 119 (80.4%) | 1506 (66.1%) | 34 (23.0%) | 110 (77.5%) |
| 2+ | 22 (15.1%) | 85 (56.7%) | 29 (19.6%) | 771 (33.9%) | 114 (77.0%) | 32 (22.5%) |
| **Condom use during last sex** |  |  |  |  |  |  |
| Yes | 49 (33.6%) | 40 (26.7%) | 37 (25.0%) | 557 (24.5%) | 47 (31.8%) | 34 (23.9%) |
| No | 97 (66.4%) | 110 (73.3%) | 111 (75.0%) | 1720 (75.5%) | 101 (68.2%) | 108 (76.1%) |
| **Intimate partner violence in past 1 year** |  |  |  |  |  |  |
| Yes | 51 (34.9%) | 25 (16.7%) | 61 (40.7%) | 809 (34.6%) | 95 (63.3%) | 88 (62.0%) |
| No | 95 (65.1%) | 125 (83.3%) | 89 (59.3%) | 1527 (65.3%) | 55 (36.7%) | 54 (38.0%) |
| **Transactional sex (past 3 months)** |  |  |  |  |  |  |
| Yes | 21 (14.4%) | 75 (50.0%) | 24 (16.2%) | 513 (22.5%) | 131 (88.5%) | 70 (49.3%) |
| No | 125 (85.6%) | 75 (50.0%) | 124 (83.8%) | 1764 (77.5%) | 17 (11.5%) | 72 (50.7%) |
| **VOICE risk score Median (IQR)** | 5 [4–6] | 4 [2–6] | 4 [2–5] | 6 [5–7] | 6 [5–7] | 4 [3–6] |
| **VOICE risk category** |  |  |  |  |  |  |
| Low (0) | 53 (36.3%) | 84 (56.0%) | 101 (68.2%) | 436 (19.1%) | 32 (21.6%) | 77 (54.2%) |
| High (1) | 93 (63.7%) | 66 (44.0%) | 47 (31.8%) | 1841 (80.9%) | 116 (78.4%) | 65 (45.8%) |
| **Any alcohol use (past 3 months)** |  |  |  |  |  |  |
| Yes | 56 (38.4%) | 34 (22.7%) | 7 (4.7%) | 1757 (77.2%) | 44 (29.7%) | 68 (47.9%) |
| No | 90 (61.6%) | 116 (77.3%) | 141 (95.3%) | 520 (22.8%) | 104 (70.3%) | 74 (52.1%) |
| **Hazardous drinking (AUDIT-C score ≥3)** |  |  |  |  |  |  |
| Yes | 56 (38.4%) | 34 (22.7%) | 7 (4.7%) | 1757 (77.2%) | 44 (29.7%) | 68 (47.9%) |
| No | 90 (61.6%) | 116 (77.3%) | 141 (95.3%) | 520 (22.8%) | 104 (70.3%) | 74 (52.1%) |
| **AUDIT-C Categories** |  |  |  |  |  |  |
| Non-drinker (score=0) | 90 (61.6%) | 116 (77.3%) | 141 (95.3%) | 520 (22.8%) | 104 (70.3%) | 74 (52.1%) |
| Low ( 1-4) | 36 (24.7%) | 26 (17.3%) | 6 (4.1%) | 849 (37.3%) | 39 (26.4%) | 33 (23.2%) |
| Medium(5-8) | 19 (13.0%) | 8 (5.3%) | 1 (0.7%) | 789 (34.7%) | 5 (3.4%) | 27 (19.0%) |
| High risk (9-12) | 1 (0.7%) | – | – | 119 (5.2%) | – | 8 (5.6%) |
| **Had alcohol with sex in past 1 month** |  |  |  |  |  |  |
| Yes | 19 (13.0%) | 15 (10.0%) | 4 (2.7%) | 963 (42.3%) | 13 (8.8%) | 54 (38.0%) |
| No | 127 (87.0%) | 135 (90.0%) | 144 (97.3%) | 1314 (57.7%) | 135 (91.2%) | 88 (62.0%) |

Note*:* P-values are not presented as sites were purposively selected, and sample sizes were highly unequal (with South Africa contributing 75% of participants). Observed differences may be a reflection of underlying sampling and geographic variation rather than random variation; geographic effects were accounted for in regression models.

**Supplementary table 2: Baseline prevalence and six months incidence of curable STIs by country among women in the INSIGHT cohort**

| **Outcome** | **eSwatini (N=146)** | **Kenya (N=150)** | **Malawi (N=148)** | **South Africa (N=2277)** | **Uganda (N=148)** | **Zambia (N=142)** |
| --- | --- | --- | --- | --- | --- | --- |
| **Prevalence at enrolment**  **n (%)** |  |  |  |  |  |  |
| Chlamydia trachomatis | 44 (30.1%) | 16 (10.7%) | 9 (6.1%) | 527 (23.1%) | 24 (16.2%) | 6 (4.2%) |
| Neisseria gonorrhoeae | 9 (6.2%) | 2 (1.3%) | 3 (2.0%) | 180 (7.9%) | 7 (4.7%) | 5 (3.5%) |
| Trichomonas vaginalis | 17 (11.6%) | 4 (2.7%) | 8 (5.4%) | 147 (6.5%) | 6 (4.1%) | 0 (0.0%) |
| Syphilis | 4 (2.7%) | 0 (0.0%) | 14 (9.5%) | 95 (4.2%) | 6 (4.1%) | 13 (9.2%) |
| ≥1 STI | 61 (41.8%) | 21 (14.0%) | 27 (18.2%) | 761 (33.4%) | 37 (25.0%) | 23 (16.2%) |
| **Incidence at month 6**  **IR per 100 PY (95% CI)** |  |  |  |  |  |  |
| Chlamydia trachomatis | 46.1 (28.0–64.1) | 5.84 (0.11–11.6) | 17.5 (7.6–27.4) | 33.8 (30.0–37.6) | 27.3 (14.3–40.3) | 15.6 (5.9–25.3) |
| Neisseria gonorrhoeae | 10.5 (2.1–18.9) | 5.84 (0.12–11.6) | 10.0 (2.6–17.5) | 11.9 (9.8–14.1) | 7.55 (0.93–14.2) | 4.56 (0.60–9.73) |
| Trichomonas vaginalis | 30.2 (15.9–44.6) | 7.33 (0.90–13.7) | 14.7 (5.6–23.8) | 11.8 (9.7–14.0) | 2.99 (–1.15–7.12) | 0.0 |
| ≥1 STI | 82.4 (57.2–108.0) | 19.6 (9.0–30.3) | 39.5 (24.0–55.0) | 54.0 (49.0–58.9) | 31.0 (17.1–45.0) | 18.9 (8.2–29.6) |

IR: Incidence rate, PY: person years , CI: confidence interval
